# Numerical Modeling of Plasmonic Nanoantennas with Realistic 3D Roughness and Distortion

**DOI:** 10.3390/s110707178

**Published:** 2011-07-13

**Authors:** Alexander V. Kildishev, Joshua D. Borneman, Kuo-Ping Chen, Vladimir P. Drachev

**Affiliations:** Birck Nanotechnology Center, School of Electrical and Computer Engineering, Purdue University, West Lafayette, IN 47907, USA; E-Mails: joshua.borneman@gmail.com (J.D.B.); kpchen@purdue.edu (K.-P.C.); vdrachev@purdue.edu (V.P.D.)

**Keywords:** optical sensing, plasmonic nanoantenna, plasmonic metamaterials, surface roughness, moving mesh, finite element method

## Abstract

Nanostructured plasmonic metamaterials, including optical nanoantenna arrays, are important for advanced optical sensing and imaging applications including surface-enhanced fluorescence, chemiluminescence, and Raman scattering. Although designs typically use ideally smooth geometries, realistic nanoantennas have nonzero roughness, which typically results in a modified enhancement factor that should be involved in their design. Herein we aim to treat roughness by introducing a realistic roughened geometry into the finite element (FE) model. Even if the roughness does not result in significant loss, it does result in a spectral shift and inhomogeneous broadening of the resonance, which could be critical when fitting the FE simulations of plasmonic nanoantennas to experiments. Moreover, the proposed approach could be applied to any model, whether mechanical, acoustic, electromagnetic, thermal, etc, in order to simulate a given roughness-generated physical phenomenon.

## 1. Introduction

Optical metamaterials arranged of plasmonic nanoantennas are important for a broad variety of applications, including near-field scanning optical microscopy (NSOM), enhanced Raman scattering, biosensors, sub-wavelength resolution imaging, and nano-scale optical lithography [1–8]. Nanoantennas rely on field enhancement due to plasmon coupling between paired metal nanostructures, such as bow-ties or paired ellipses [3,6,9–13], strips [8,14,15], rods [1,5,16–18], and other structures. The field enhancement is a key parameter for sensing applications. It is affected by the material quality and nanostructure geometry, including the non-ideality of both. In order to properly design these structures for a specific performance, and for post-fabrication retrieval of optical properties, simulations are normally used, including finite element methods [8,12,19]. Note that plasmon coupling between particles in quasi-regular network assembled with DNA and used for cancer cell profiling [20] is based on similar physics and also must be designed with a realistic set of material parameters and geometry.

Absorption (loss) in a nanoantenna system comes from a variety of sources, typically including internal grain boundaries in the metal antennas [21], chemical interface effect, and surface roughness. All of these factors could be size dependent [22]. A single “loss factor” [19] is sufficient to match most experimental results, but does not distinguish between these effects, nor convey the relative importance of each. By simulating both ideal (smooth) and rough antennas with otherwise identical material properties, we may observe the role which roughness plays on nanoantenna performance and loss. The goal is to separate the effect of geometrical roughness from metal permittivity changes due to internal grain boundaries and size effects. This modeling will help to compare the effect of shape changes for each particle with the changes in the gap size between two particles of a nanoantenna. Also, a methodology for statistically defined roughness modeling has been developed here. Overall this method will make nanoantenna designs and simulations more predictive.

**Figure 1.**
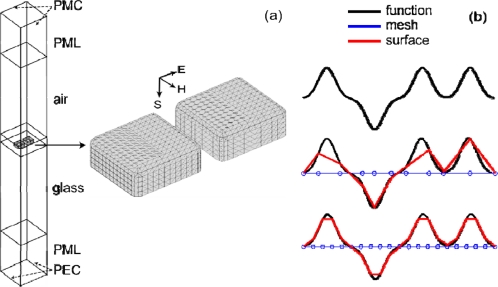
**(a)** Schematic of a nanoantenna unit cell. The perfect magnetic conductor (PMC) and the perfect electric conductor (PEC) boundary conditions are used to account for the symmetry of the double-periodic array at normal incidence. Perfectly matched layers (PML) are used prevent the reflection of the incident and scattered light from the top and bottom ends of the FE domain. Primary (P) polarization is shown; **(b)** A fine mapped mesh at the distorted interfaces is required to reproduce all the statistically equivalent realizations of a given roughness with high accuracy.

Note that the effect of roughness on surface plasmon polariton propagation along a large area interface [23], as well as on the plasmon resonance of single spherical [24] and nanorod particles [25], has been studied experimentally and through modeling. Here, nanostructures based on an interparticle plasmon coupling are under study. It was shown earlier that the roughness in such systems affect the performance of nanoantennas [21] and metamagnetics [22]. The roughness geometry was modeled similar to the electron microscopy images of real samples [21]. The complete 3D surface roughness approach used here is more realistic than earlier works using simplified 2D cases. We analyze the effect of surface roughness using a statistically defined distortion of a nanoantenna geometry. By using a moving 3D finite-element method (FEM) mesh, we preserve the degrees of freedom (DOF) number of the original “smooth” model and simply morph the structure of the mesh to accommodate the moving boundary. A typical set-up for the nanoantenna array simulation using FEM is depicted in Figure 1. Hence, the specific aim of the paper is to show the efficiency of this approach based on a moving mesh in FEM nanoantenna models.

In general, the moving mesh models (MMM) of roughness entail a mapping from ideally smooth interfaces between the domains of elemental materials in an initial (ideal) space to an irregularly shaped interface in realistic physical space. This is done by connecting the discrete points of physical interfaces with corresponding discrete points of ideally smooth boundaries, while the interior of both the physical and the ideal domains are covered with an appropriate 3D FE mesh, which in our particular case, are suitable for the solution of monochromatic 3D Maxwell’s equations.

The key components of our MMM include:

- *the stochastic roughness equations,* which determines a one-to-one mapping from ideal interfaces in an initial space to irregularly shaped interfaces in physical space. Choosing simple yet realistic roughness equations and being able to solve them efficiently are very crucial for MMM. The roughness equations we use are based on a finite number of 2D Gaussian surface distortions distributed over the surface that can be written as follows:
  (1) $$\tilde{z}(\tilde{x},\tilde{y})={\tilde{z}}_{0}+\sum_{n=1}^{N}d{\tilde{z}}_{n}(\tilde{x},\tilde{y})=\sum_{n=1}^{N}a_{n}\;\text{exp}\;\left[-{\left(\frac{\tilde{x}-{\tilde{x}}_{n}}{\sigma_{n}}\right)}^{2}\right]\;\text{exp}\;\left[-{\left(\frac{\tilde{y}-{\tilde{y}}_{n}}{\sigma_{n}}\right)}^{2}\right]$$
  where the *z̃*-component of the local coordinate system (*x̃, ỹ, z̃*) is aligned with the external surface normal vector, *z̃*_0_ is a constant level, *dz̃_n_* (*x̃*, *ỹ*) is the local displacement due to the *n*^th^ Gaussian distortion, *a_n_* is the magnitude is and *σ_n_* is the full-width half-maximum (FWHM) of the *n*^th^ distortion at (*x̃_n_*, *ỹ_n_*), and *N* is the total number of surface distortions:
- *the roughness quality estimates,* which are used for assessing the quality of the distorted mesh for approximating given roughness features. It may depend on the desired curvature, and spatial distribution of the Gaussian distortions. In practice, a more dense and uniform surface mesh is always desirable as shown in Figure 1(b).
- *sets of statistically equivalent realizations and their averaging,* which are employed to implement different realizations of rough antennas using statistically equivalent roughness equations; then, averaging of the far-field spectral optical responses is necessary. In particular, we have used RMS averaging of the spectra within each set of equivalent realizations.

Using the above components, a comparison of the resulting simulated transmission and reflection spectra for smooth nanoantennas versus roughened nanoantennas is presented and examined.

## 2. Results and Discussion

### 2.1. Method

This study, first applies a moving mesh model in order to map a statistically defined roughness onto the surface of the nanoantennas in a user-controlled manner. In our case specifically, the mesh on the flat surfaces of the nanoantennas are displaced using 48 2D-Gaussian bumps with a defined amplitude of either positive (bump out) or negative (bump in) values, and a defined full-width half-max (FWHM). There are an equal number of “in” and “out” bumps to preserve the original volume of the nanoantenna, although they are spread randomly over the surface. A moving mesh solver is initially used, leading to a model with roughened surfaces. Both the original “smooth” mesh, and the displaced “rough” mesh are saved and loaded into a separate electromagnetics model. Rearranging the bump profiles over the surface of the nanoantenna is done simply by indexing the array of bump amplitudes or “rotating” the bumps, leading to different roughness profiles, but with the same statistical qualities.

Nanoantenna FEM models are typically meshed using a “normal” quality free-mesh. In this study we use a mapped mesh on the nanoantenna surface so that when the bumps are rearranged to different locations, each bump will maintain the same profile regardless of its “rotated” or rearranged position. A free mesh, which is nonuniform over the surface, would cause a single bump with a constant definition to appear different depending on the mesh where it is placed. This approach allows us to properly model the effect of surface roughness on nanoantenna performance.

Due the symmetry of a smooth nanoantenna unit cell, typically only a quarter unit-cell is simulated, however our model uses a full unit cell. This is done so that separate roughness profiles may be placed on each particle of the nanoantenna pair in order to prevent symmetric peak resonances across the gap.

A typical smooth nanoantenna array unit cell is shown in Figure 1, where the surface of the nanoantennas use a mapped mesh, and materials and simulation boundary conditions are labeled. The grid size of the mapped mesh is defined by setting a maximum element size for each edge, and is set to be one-quarter of the bump FWHM. This mesh size was sufficient to capture the salient features of each bump and gives an accurate modified gap spacing due to each roughness feature. Test simulations with a further reduction in the mesh size did not result in statistically significant differences, while greatly increasing the simulation time due to the increased number of degrees of freedom in the model. Free meshes are used for the air and glass regions.

**Figure 2.**
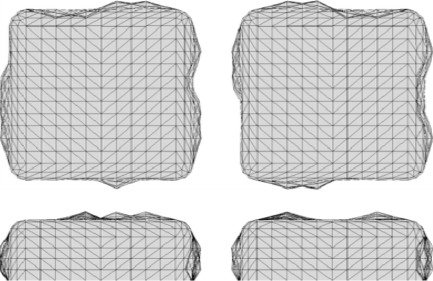
Example nanoantenna roughness iteration 1.

**Figure 3.**
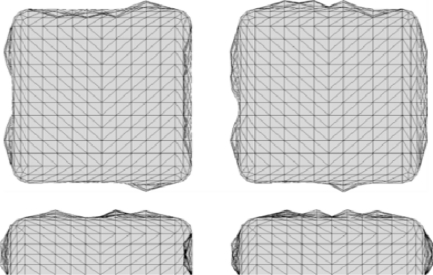
Example nanoantenna roughness iteration 2.

Examples of the resulting roughened nanoantennas are shown in Figures 2 and 3 for two different roughness arrangements. In the example shown here, nanoantennas were modeled with a unit cell size of 400 nm by 400 nm. Their x, y dimensions are 108 nm by 102 nm respectively with a thickness of 36 nm. The gap between the two nanoantennas is 28 nm. The nanoantennas material permittivity is modeled using a Drude-Lorentz model [19] for gold with a loss factor of 1.3. To understand the effect of roughness on the electromagnetic response of these nanoantennas, roughness profiles with amplitudes of both ±5 nm and ±10 nm, and a full-width half-max *s_n_* of 20 nm (see Equation (1)), are compared to the smooth nanoantenna model.

One should mention that the surface roughness depends on many factors. Specifically it depends on the nanostrusture size, substrate material, and fabrication method. A very low surface roughness, 0.65 nm for a test large area Ag film and about 2 nm for patterned metal layers, was reported recently [23]. This achieved low roughness is due to the high surface quality of the Si substrate and the fact that the metal deposition was done in the form of large area layers. These conditions can be very different from metal deposition on a photoresist pattern made by e-beam lithography and followed by a lift off process. In the last case the roughness can be up to several nanometers. We believe that our choice of surface roughness at 0, 5, and 10 nm covers an important range from very smooth to 10 nm. The presented model cannot be universal of course, but this model allows for easy adjustment to the number of bumps and their height depending on specifically fabricated nanoantennas, in addition to material changes to the nanoantennas and substrate.

Electromagnetics are then simulated with boundary conditions and a primary (P) E-field polarization as shown in Figure 1, and a secondary polarization (S) with the field rotated 90° (and an appropriate switching of boundary conditions), and then solved using a commercially available solver (COMSOL Multiphysics). The electromagnetics model consist of four multiphysics models, two models for each polarization: one with all materials set to air as a reference, and one with the proper nanoantenna materials. All the models use the same geometry and mesh. The reference field is used to calculate transmission and reflection of the nanoantenna models, and was found to be identical regardless of mesh displacement. Therefore, in order to save simulation time, the reference fields were only calculated once.

To illustrate the computational complexity required to simulate a roughened structure, the final mesh in our example contains about 48 thousand elements, giving each of the four electromagnetics models 943,000 DOF. These simulations were ran on a single cluster node with 2 Quad-Core Intel E5410 2.33 GHz processors and 16 GB of memory. Solution times varied, but averaged around 9 h for each model simulated at 9 wavelengths.

### 2.2. Results

By comparing the far-field transmission and reflection spectra for the smooth mesh model (an ideal nanoantenna) to the spectra for various arrangements of roughness (a rough nanoantenna), we may determine the effects of surface roughness on the electromagnetic performance of the nanoantennas. Specifically, we may isolate the effect of geometric surface roughness from other effects due to internal material properties on the plasmonic performance of the nanoantennas.

**Figure 4.**
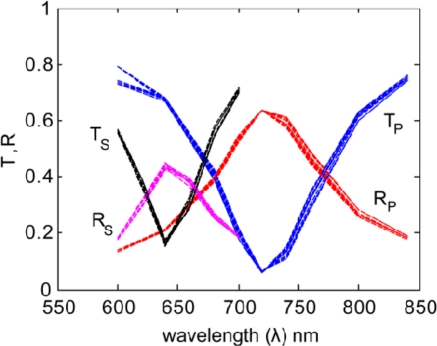
Transmission and reflection spectra for the primary (P) and secondary (S) polarizations for statistically equivalent 5-nm roughness realizations.

**Figure 5.**
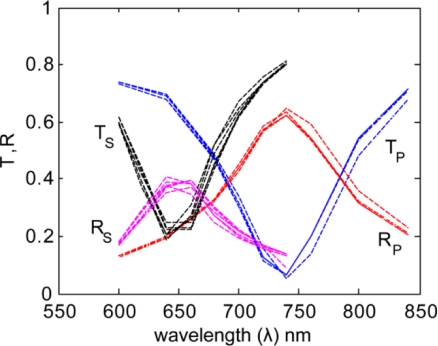
Transmission and reflection spectra for the primary (P) and secondary (S) polarizations for statistically equivalent 10-nm roughness realizations.

In Figures 4 and 5 we show the results for several realizations (roughness arrangements) of each roughness profile (5 nm and 10 nm bump amplitudes). These multiple iterations were averaged to represent the results from an array of many nanoantennas, as is present in an actual sample.

**Figure 6.**
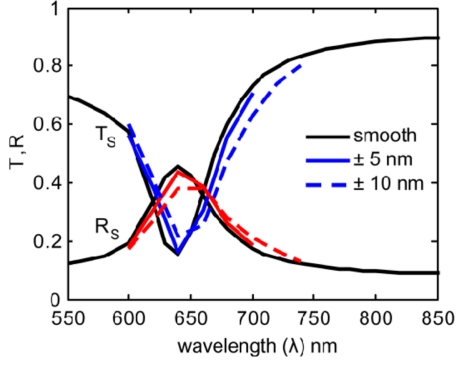
Spectra for the secondary (S) polarization for smooth (black), 5-nm roughness (cyan), and 10-nm roughness (cyan-dash).

**Figure 7.**
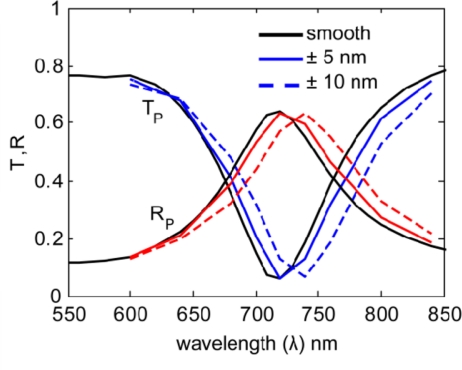
Spectra for the primary (P) polarization for smooth (black), 5-nm roughness (blue), and 10-nm roughness (blue-dash).

The resulting averaged spectra are shown in Figures 6 and 7. By comparing the smooth nanoantenna model with the results from each of the rough models, we see that surface roughness does not significantly increase the magnitude of the resonant absorption, but instead produces a shift in the resonance wavelength. This might be due to the roughness inducing a variation in the width of the gap, which will affect the resonant wavelength. It is important to note that while the loss-factor modeling parameter, a single value typically incorporating roughness along with many other physical material properties and discussed elsewhere [12,21,22], usually results in a strong decrease in the strength of the resonance, roughness alone does not significantly reduce the resonance, but does result in a shift of wavelength. This means that the effects of surface roughness, which are typically incorporated into loss-factor, may be distinguished separately if the model properly accounts for surface roughness, as has been done here.

Fitting the results with Lorentz curves allows us to retrieve various metrics from the spectra. The results, shown in Table 1, indicate that the implementation of surface roughness leads to a significant shift in the primary resonance wavelength (P-peak).

## 3. Conclusions

Optical plasmonic nanoantennas are important for advanced optical sensing and imaging applications including surface-enhanced fluorescence, chemiluminescence, and Raman scattering. Realistic nanoantennas have nonzero surface roughness and are different from bulk metal quality, which will affect the resulting enhancement factor. These issues should be accurately accounted for in the nanoantenna design. To the best of our knowledge, this work is the very first attempt to directly model the effect of realistic 3D surface roughness in nanoantenna arrays. Instead of regenerating the mesh for each roughness realization, a moving mesh is used to apply roughness, therefore preserving the total number of elements. In the case we consider, the introduction of roughness into the nanoantenna model does not result in significant loss, but does result in a shift in the resonance wavelength. This result can be critical when fitting simulations to experiments. Nanoantennas designed for a narrow resonance wavelength, and a particular sensor application, may need to account for this realistic fabrication induced shift away from the ideal geometric performance, in order to optimize the design. The proposed method has much wider applications that go beyond numerical modeling of the optical responses of nanoantenna arrays or plasmonic metamaterials. Most physical models make assumptions that surfaces are smooth; however the actual roughness may have a significant effect on the simulated physical processes and therefore on the accuracy of the modeling results. This method may be applied to any model, whether mechanical, acoustic, electromagnetic, thermal, *etc.*, in order to model the effects of roughness on the results and simulate a roughness-generated physical phenomenon.

## Figures and Tables

**Table 1.** Metrics for the primary (P) and secondary (S) polarization spectra. Resonance wavelength (peak) and full-width half-max (width) in nanometers.

**(nm)**	**Smooth**	**±5 nm**	**±10 nm**
**P-peak**	720 (±3)	724 (±4)	738 (±4)
**P-width**	48 (±3)	52 (±5)	54 (±5)
**S-peak**	638 (±3)	646 (±4)	647 (±4)
**S-width**	35 (±3)	33 (±10)	43 (±3)
